# PROX1-associated lymphatic reprogramming signatures in pediatric adamantinomatous craniopharyngioma: a comparative study with adult cases

**DOI:** 10.3389/fped.2026.1865684

**Published:** 2026-09-16

**Authors:** Wanyu Zhang, Dingkang Xu, Dengpan Song, Qiang Gao

**Affiliations:** 1 Department of Rheumatology and Immunology, Beijing Children’s Hospital, Capital Medical University, National Center for Children’s Health, Beijing, China; 2 Department of Neurosurgery, Guangdong Provincial People’s Hospital (Guangdong Academy of Medical Sciences), Southern Medical University, Guangzhou, China; 3 Department of Neurosurgery, The First Affiliated Hospital of Zhengzhou University, Zhengzhou, China; 4 Department of Neurosurgery, Beijing Children’s Hospital, Capital Medical University, National Center for Children’s Health, Beijing, China

**Keywords:** lymphatic reprogramming, LYVE1, pediatric adamantinomatous craniopharyngioma, PROX1, tumor microenvironment

## Abstract

**Background:**

Adamantinomatous craniopharyngioma (ACP) is the most common sellar tumor in children. Although histologically benign, pediatric ACP often exhibits marked local invasiveness and a high postoperative recurrence rate. Emerging evidence suggests that the tumor microenvironment (TME), particularly immune infiltration and lymphatic-related structures, may play an important role in ACP progression. However, the presence and clinical relevance of lymphatic reprogramming in ACP remain poorly understood.

**Methods:**

Publicly available single-cell RNA sequencing data from a pediatric ACP specimen were analyzed to characterize cellular composition and PROX1 expression patterns. Immunohistochemistry was subsequently performed on pediatric and adult ACP tissues to examine the spatial distribution and age-related expression of lymphatic markers, including PROX1 and LYVE1, as well as the vascular endothelial marker CD34. Quantitative comparisons between pediatric and adult cohorts were conducted.

**Results:**

Single-cell analysis identified ten major cell populations within pediatric ACP, with epithelial cells accounting for approximately 40% of total PROX1 expression. Immunohistochemical validation revealed strong PROX1 expression predominantly localized to palisade-like and keratinized epithelial regions, while glial scar areas were largely negative. LYVE1-positive lymphatic-like structures were observed within the tumor parenchyma and showed partial spatial overlap with CD34-positive vascular structures, supporting the presence of PROX1-mediated lymphatic reprogramming. Notably, PROX1 and LYVE1 expression levels were significantly higher in pediatric ACP than in adult cases, indicating a marked age-dependent difference.

**Conclusions:**

Our findings demonstrate the presence of lymphatic-like structures characterized by PROX1 and LYVE1 expression in pediatric ACP, indicating that lymphatic-like reprogramming is associated with the tumor microenvironment. These features could potentially relate to the aggressive and infiltrative clinical behavior observed in pediatric cases. Therefore, exploring the modulation of PROX1-driven pathways warrants further investigation as a potential approach for pediatric ACP.

## Introduction

Adamantinomatous craniopharyngioma (ACP) is the most common type of sellar region tumor in children. Although this tumor is intracranial, many consider this tumor to be extra-axial to the central nervous system (1–3). Although classified as a benign neoplasm based on histopathological criteria, ACP frequently exhibits pronounced local invasiveness and a high rate of postoperative recurrence, resulting in suboptimal long-term clinical outcomes (4, 5). ACP has a bimodal age distribution; it manifests in children, accounting for 5%–11% of childhood intracranial tumors and later in adulthood between 45 and 60 years of age, often more indolent clinical presentation and prognosis in adults (6). In clinical practice, surgical strategies often prioritize avoidance of irreversible injury to the posterior hypothalamus, which may necessitate subtotal resection and consequently increase the risk of tumor recurrence. Postoperative radiotherapy is therefore commonly employed to suppress tumor regrowth (7).

Recent studies have highlighted the highly heterogeneous tumor microenvironment (TME) of ACP, in which immune cell infiltration and lymphatic-related structures are increasingly recognized as important contributors to tumor initiation and progression (8, 9). The discovery and functional characterization of the intracranial lymphatic system have provided new insights into the pathophysiology of neurological diseases. Lymphatic-like structures have been identified in a variety of central nervous system (CNS) disorders, with the transcription factor PROX1, a master regulator of lymphatic endothelial cell fate, and the lymphatic vessel endothelial hyaluronan receptor 1 (LYVE1) widely used as canonical lymphatic markers (10).

Accumulating evidence suggests that the expression profiles of these lymphatic-associated molecules vary significantly across age groups, potentially accounting for age-dependent differences in tumor biology (10). Beyond its role in lymphatic development and maintenance, PROX1 may cooperate with the Wnt/β-catenin signaling axis—the central oncogenic pathway in ACP—to promote tumor progression (11, 12). Within the lymphatic system, PROX1 acts in concert with connexin 45 (Cx45) to maintain structural and functional homeostasis (13). In addition, LYVE1-positive macrophage subsets have been shown to exert dual roles in inflammatory regulation and may facilitate tumor-associated lymphangiogenesis through the secretion of VEGF-C and other factors (14). PROX1 and LYVE1 have also been applied to evaluate CNS dissemination potential in primary CNS lymphoma and diffuse large B-cell lymphoma (15). Clinically, pediatric ACP is widely regarded as more invasive than its adult counterpart, a feature that may be closely linked to the distribution and activity of lymphatic-like structures within the tumor.

Based on single-cell RNA sequencing analysis of one pediatric ACP specimen (16), we observed marked upregulation of PROX1 in tumor epithelial cells, suggesting active lymphatic generation or remodeling within ACP lesions. Building upon this observation, we performed immunohistochemical analyses on pediatric and adult ACP tissues to systematically characterize the age-specific distribution of lymphatic markers and to explore their associations with clinicopathological parameters (17, 18). Our study aims to provide a theoretical basis for the development of therapeutic strategies targeting pro-lymphangiogenic pathways in pediatric ACP.

## Methods

### Patient recruitment and data collection

This study involved a cohort of 6 pediatric and 6 adult patients with ACP, recruited from Beijing Children's Hospital and the First Affiliated Hospital of Zhengzhou University between July 1, 2024, and December 30, 2025. Comprehensive clinical and pathological data were collected, including age, gender, clinical symptoms, tumor size, location, and prognosis. The study protocol was approved by the Ethics Committees of both participating institutions, and written informed consent was obtained from all participants prior to enrollment.

### Single-cell RNA sequencing analysis

To investigate the cellular landscape and gene expression profiles, we utilized publicly available single-cell RNA sequencing data from an ACP sample (GSM6265813, https://sra-pub-run-odp.s3.amazonaws.com/sra/SRR19646669/SRR19646669) (16). Specifically, the expression levels of PROX1 were quantified and analyzed according to the methodology previously established in the literature (19).

#### Single-nuclei RNA sequencing (snRNA-seq) tissue preparation and library construction

Fresh tissue was surgically dissected and immediately flash-frozen in liquid nitrogen. Nuclei were isolated following the 10× Genomics demonstrated protocol (CG000375, Rev A). On the day of the experiment, frozen tissue was homogenized in NP40 Lysis Buffer supplemented with Protector RNase Inhibitor (Sigma-Aldrich) and filtered through a 70 µm cell strainer (Falcon). The filtrate was centrifuged at 500 × g for 5 min at 4 °C to pellet nuclei, which were then resuspended in wash buffer. This suspension underwent a second round of straining and centrifugation under identical conditions. Washed nuclei were resuspended in wash buffer and stained with 7-AAD (SML1633, Sigma-Aldrich) for fluorescence-activated cell sorting (FACS).

Purified nuclei were collected by centrifugation at 500 × g for 6 min and resuspended in wash buffer to a target concentration of 900 nuclei/µL. A total of 15 µL of this single-nuclei suspension (∼13,500 nuclei) was loaded into one channel of a Chromium Single Cell G Chip (10× Genomics, 1000120) to achieve an expected recovery of 8,000–9,000 nuclei. Single-nuclei barcoding, cDNA synthesis, and library preparation were performed using the Chromium Single Cell 3′ Library & Gel Bead Kit v3.1 (10× Genomics, 1000121) according to the manufacturer's instructions (Single Cell 3′ Reagent Kits User Guide, Version 3.1). Finally, libraries were sequenced on the Illumina NovaSeq 6000 platform in a 2 × 150 bp paired-end configuration, yielding approximately 300 million reads per library.

#### snRNA-seq data processing and downstream analysis

Raw sequencing reads were aligned to the human reference genome (hg38), and initial cell calling as well as gene expression matrix generation were performed using the cellranger count function (Cell Ranger software suite, v6.0.1) with default parameters.

Downstream bioinformatic analysis was conducted using the R package Seurat (v3.0.2). To remove low-quality data and potential doublets, nuclei with fewer than 800 or more than 7,500 detected transcripts (UMIs) were filtered out. Filtered expression matrices were normalized using NormalizeData, and 2,000 highly variable features were identified using FindVariableFeatures. Linear transformation and data scaling were performed via ScaleData, followed by Principal Component Analysis (PCA) via RunPCA for dimensionality reduction. The dimensionality of the dataset was evaluated using ElbowPlot, and the top 20 principal components (PCs) were selected for downstream clustering. Nuclei were clustered using FindNeighbors and FindClusters with a resolution parameter of 0.4. For visualization, Uniform Manifold Approximation and Projection (UMAP) was applied using RunUMAP based on the first 20 PCs.

To evaluate cell-type-specific transcriptional profiles within the tumor microenvironment, the expression levels of PROX1 were systematically analyzed across all annotated cell clusters. The percentage of PROX1-positive cells and its average expression intensity within each cluster were quantified and visualized using dot plots and feature plots in Seurat.

### Histopathological and immunohistochemical examination

Formalin-fixed, paraffin-embedded (FFPE) tissue sections were subjected to Hematoxylin and Eosin (HE) and immunohistochemical (IHC) staining, following previously described protocols (20). Tissue sections were processed using the Leica Bond-MAX automated staining system (Leica Biosystems, Wetzlar, Germany). For IHC, primary antibodies against PROX1 (Rabbit mAb, ABclonal, Wuhan, China), Nestin (Rabbit mAb, ABclonal), CD34 (Rabbit PolymAb, ABclonal), and LYVE1 (Rabbit mAb, ABclonal) were employed, followed by incubation with appropriate secondary antibodies (Zhongshan Golden Bridge Biotechnology, Beijing, China). Quantitative IHC analysis was performed using Image J (v1.53) across three randomly selected fields per sample. Total cell numbers were based on nuclear counts, with a positive cell defined by specific immunostaining adjacent to the nucleus. All scoring was conducted by an investigator blinded to group allocation. The PROX1 and LYVE1 positivity rate for each sample was defined as the mean percentage of positive cells across the three analyzed regions.

### Statistical analysis

Data analysis was performed using SPSS version 22.0. Quantitative results are presented as the mean ± standard error of the mean (SEM). Differences between two groups were evaluated using the non-parametric Mann–Whitney *U* test. *P* < 0.05 was considered statistically significant.

## Results

### Single-cell landscape of pediatric ACP and PROX1 expression

To investigate the role of lymphatic components in craniopharyngiomas, we performed single-cell RNA sequencing (scRNA-seq) to profile the cellular landscape and evaluate the expression of the key lymphangiogenic factor, PROX1. Analysis of publicly available pediatric ACP data identified ten major cell populations, including T cells, B cells, fibroblasts, macrophages, astrocytes, epithelial cells, endothelial cells, neurons, and oligodendrocyte precursor cells (OPCs) (Figure 1A). The relative proportions of these clusters are consistent with previous reports and highlight the robust inflammatory microenvironment characteristic of ACP (19). Although epithelial cells represent a smaller fraction of the total population, they serve as the primary drivers of tumorigenesis and pathological architecture, maintaining a distinct transcriptional profile. Notably, while PROX1 was detected across multiple clusters, the epithelial compartment accounted for nearly 40% of its total expression, suggesting these cells play a central role in modulating lymphangiogenic signaling within the tumor (Figure 1B).

**Figure 1:**
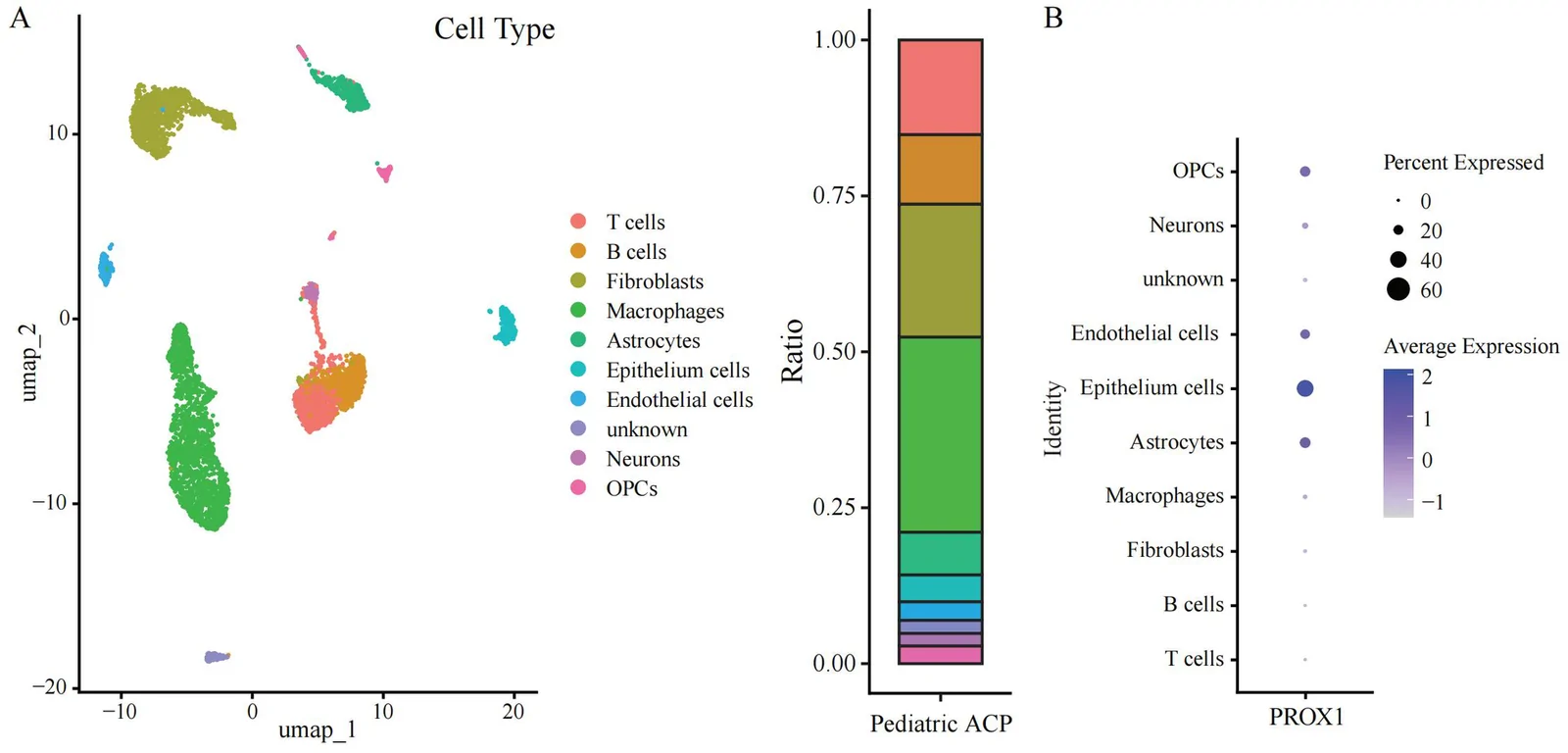
Single-nuclei transcriptomic landscape of pediatric ACP tissue. **(A)** UMAP plot displaying the major cell populations identified in a pediatric ACP tissue, including T cells, B cells, fibroblasts, macrophages, astrocytes, epithelial cells, endothelial cells, neurons, and OPCs, along with their relative proportions. **(B)** Cell-type-specific expression pattern of PROX1 across the identified cell clusters.

### Spatial localization of PROX1 in pediatric ACP tissues

To validate these findings, histopathological and immunohistochemical analyses were performed on additional pediatric ACP specimens. Hematoxylin and eosin staining revealed classic pathological features, including palisading epithelium and wet keratin (Figure 2A). Consistent with prior descriptions, tumor tissues could be subdivided into whorl-like epithelium (WE), palisade-like epithelium (PE), keratinized-like epithelium (KE), and glial scar regions (Figure 2B).

**Figure 2:**
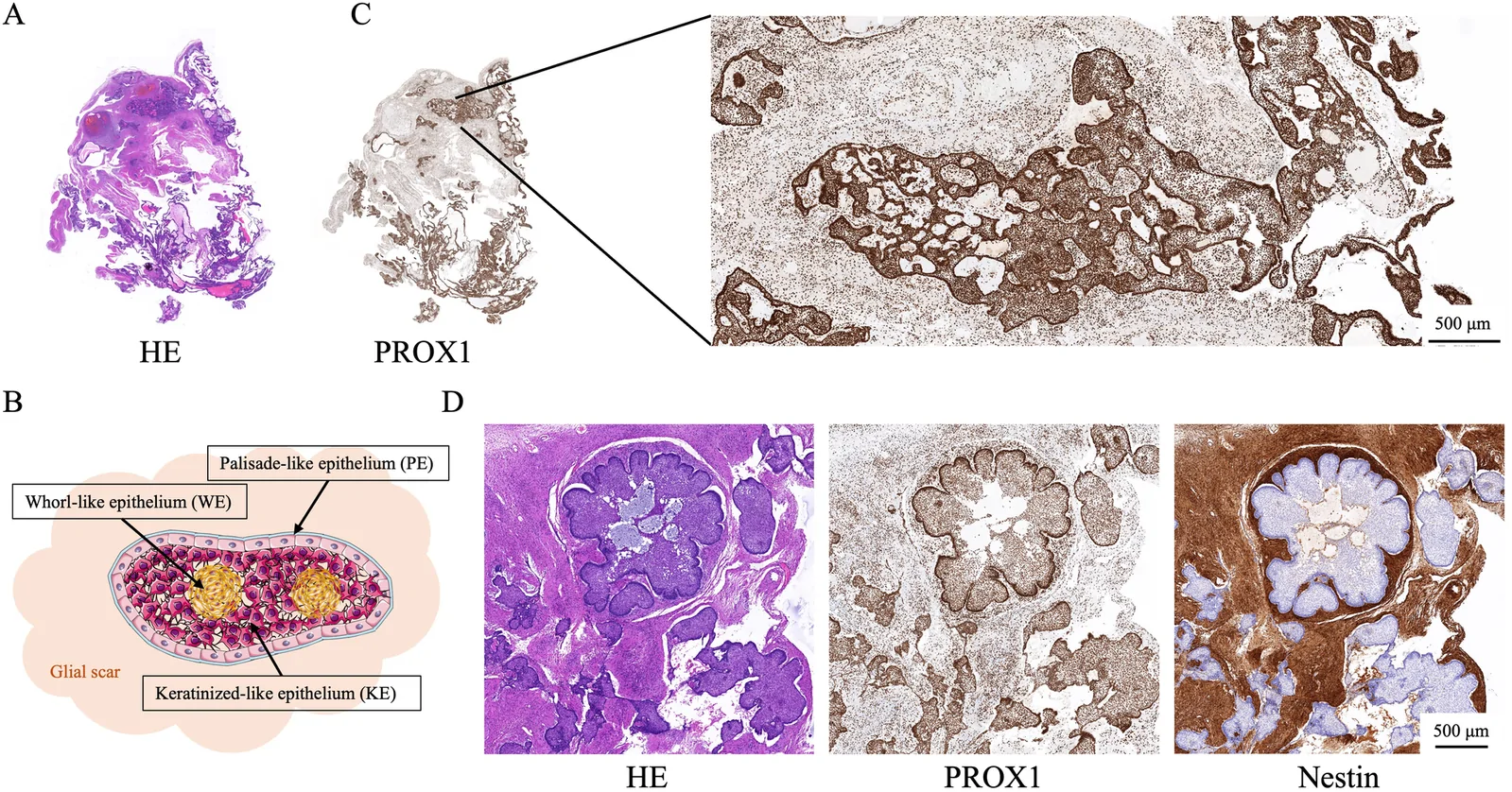
Characterization of the pathological landscape and cellular distribution in pediatric ACP. **(A)** HE staining revealing typical histopathological abnormalities. **(B)** IHC staining delineating the topographic distribution of PROX1 within the pediatric ACP. **(C)** A conceptual model depicting the spatial organization of key neoplastic cell states, including Palisade-like epithelium (PE), Whorl-like epithelium (WE), Keratinized-like epithelium (KE) and Glial scar. **(D)** Comparative HE and IHC analysis defining the spatial relationship between PROX1 and Nestin expression. Scale bar, 500 µm.

**Figure 3:**
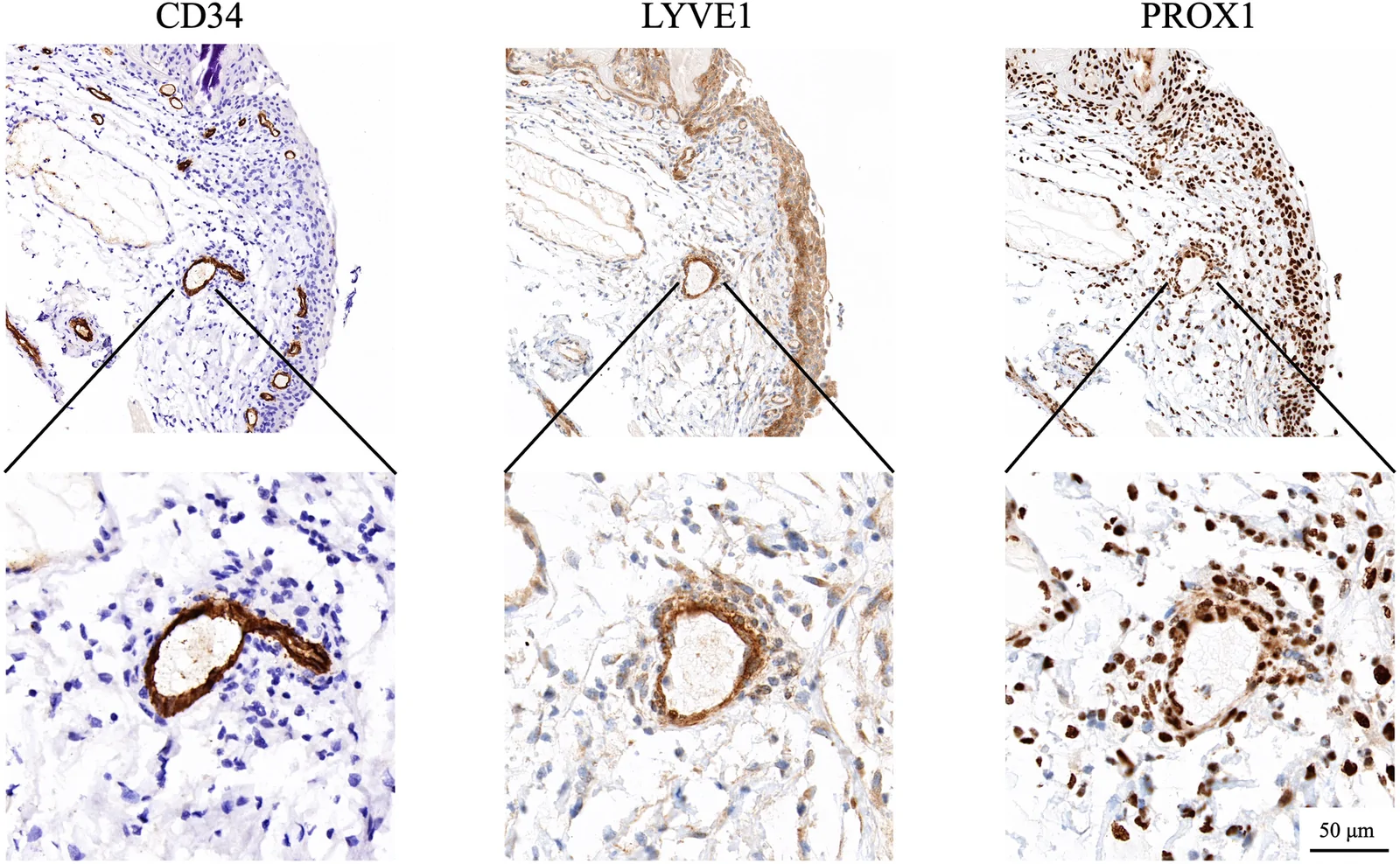
Spatial distribution and co-expression of endothelial and lymphatic markers in pediatric ACP tissues. Representative IHC staining of serial sections from pediatric ACP tissues demonstrating the spatial distribution of CD34, PROX1, and LYVE1. All three markers display overlapping expression that clearly delineates the same ring-like structures, indicating a distinct lymphatic phenotype within the tumor microenvironment. Scale bar, 50 µm.

**Figure 4:**
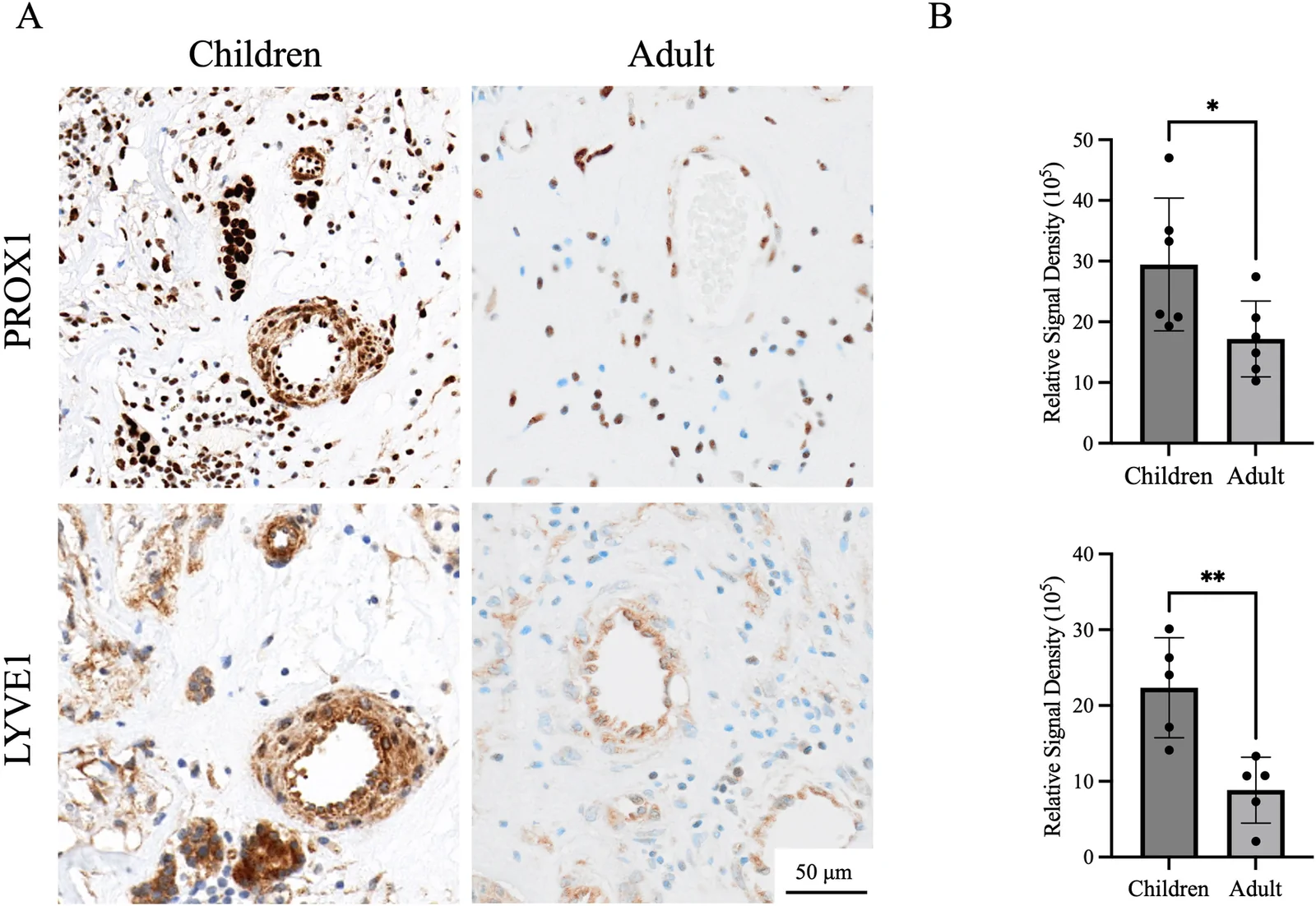
Comparative analysis of PROX1 and LYVE1 expression in children versus adult ACP. **(A)** Representative IHC staining delineating the spatial distribution of PROX1 and LYVE1 in pediatric and adult ACP tissues. **(B)** Quantitative analysis showing the differential expression of PROX1 (*n* = 6) and LYVE1 (*n* = 5) between pediatric and adult cohorts. Scale bar, 50 µm. *: *P* < 0.05; **: *P* < 0.01.

Immunohistochemistry demonstrated that PROX1 protein was predominantly localized within PE and KE regions, whereas non-epithelial areas exhibited minimal expression. This distribution closely mirrored the single-cell transcriptomic findings (Figure 2C). Analysis of adjacent sections revealed a mutually exclusive spatial pattern between PROX1 and the glial scar marker Nestin, indicating an absence of PROX1 expression within peritumoral gliotic zones. High PROX1 expression within epithelial cores suggests enhanced invasive potential and a tendency toward lymphatic-like differentiation (Figure 2D).

### Evidence of lymphatic reprogramming and age-dependent differences

We next examined the expression of the vascular endothelial marker CD34 and the lymphatic endothelial marker LYVE1 using immunohistochemistry on adjacent sections. Distinct LYVE1-positive lymphatic-like structures were observed within the tumor parenchyma of pediatric ACP, partially overlapping with CD34-positive vascular structures (Figure 3). These findings support the hypothesis of PROX1-mediated endothelial-to-lymphatic reprogramming within the pediatric ACP microenvironment (21, 22).

Given the bimodal age distribution of ACP, we further analyzed adult ACP specimens using the same immunohistochemical approach. In contrast to pediatric cases, adult ACP tissues exhibited minimal PROX1 and LYVE1 expression. Quantitative analysis revealed significantly higher PROX1 (*P* = 0.0382) and LYVE1 (*P* = 0.0051) expression levels in pediatric ACP, with LYVE1 expression reaching approximately twofold that of adult tumors (Figure 4). These age-related differences suggest that lymphatic reprogramming may underlie the distinct clinical behavior of pediatric ACP and contribute to its heightened invasiveness (23).

## Discussion

The CNS has long been regarded as an “immune-privileged” organ lacking a classical lymphatic drainage system. In this study, we identified lymphatic-like structures expressing PROX1 and LYVE1 within pediatric ACP tissues, providing evidence that this histologically benign tumor may establish a unique transport and signaling network through lymphatic reprogramming. Such structures may facilitate the dissemination of tumor-derived inflammatory mediators, including senescence-associated secretory phenotype (SASP) factors, into surrounding brain parenchyma, thereby offering a plausible explanation for the frequent hypothalamic involvement and diffuse infiltration observed in pediatric ACP.

By comparative analysis of pediatric and adult specimens, we observed an upregulation of PROX1 and LYVE1 in pediatric ACP, reflecting characteristics that warrant further validation as specific lymphatic markers. These findings provide preliminary molecular clues that may correlate with the clinically observed aggressive phenotype of pediatric ACP. It is conceivable that PROX1-driven microenvironmental remodeling potentially influences intertumoral fluid dynamics and immune cell trafficking, which warrants further investigation regarding its role in enhanced invasive capacity. The observed age-associated variation hints at the possibility that ACP exhibits distinct microenvironmental features across different age groups, reflecting the potential impact of host brain developmental stages.

Given the cooperative interaction between PROX1 and the Wnt/β-catenin pathway, the PROX1-high epithelial regions (PE and KE) identified in this study are likely to represent the leading invasive fronts of the tumor. Therapeutic options for recurrent ACP remain limited. Anti-VEGF therapy with bevacizumab has shown encouraging results in adult ACP (24), but its use as a standalone treatment in pediatric patients remains uncommon (25). As a central regulator of lymphatic reprogramming, early inhibition of PROX1 prior to neovascular formation may represent a potential strategy to restrain pediatric ACP progression.

All-trans retinoic acid (ATRA), a clinically approved agent known to modulate lymphatic development, has been shown to suppress the growth of ACP models *in vitro* (26–28). In addition, bleomycin, a sclerosing agent widely used in the treatment of lymphatic malformations, was proposed as an intracystic therapy for ACP as early as 1985 by Takahashi (29). Subsequent small-scale studies confirmed that intracystic bleomycin could induce sustained tumor shrinkage in pediatric ACP, with complete response rates ranging from 29% to 67% and durable effects maintained over a mean follow-up period of less than seven years (30–37). Given the involvement of lymphatic-like features in ACP, these clinical and experimental observations lead us to hypothesize that targeting lymphatic endothelial biology or vessel density might represent a potential therapeutic avenue for ACP, a concept that warrants rigorous future functional and preclinical validation (38), Dong Hyuk Park et al. indicated that approximately 30% of craniopharyngioma patients experience transient toxic reactions following post-operative bleomycin injection (35). Additionally, a report by Mette Linnert et al. noted that severe adverse events occurred in 3% of brain tumor patients, which were closely associated with high-dose bleomycin administration (39). Future clinical paradigms should prioritize age-based patient stratification to deliver tailored therapeutic regimens, leverage spatial transcriptomics or proteomics for unbiased analysis of tumours and the TME, and emphasize the critical role of genetic typing (40). Furthermore, incorporating family-centered care into clinical management may also significantly improve pediatric patient outcomes (41). In this context, pharmacological targeting of the PROX1 signaling axis represents a novel and potent strategy to mitigate the aggressive phenotype in children with ACP.

## Data Availability

The single-nuclei RNA sequencing data supporting the findings of this study have been deposited in the NCBI Gene Expression Omnibus (GEO) database and are publicly accessible under accession number GSM6265813 (https://www.ncbi.nlm.nih.gov/geo/query/acc.cgi?acc=GSM6265813).
